# Phase Synchrony Analysis of Rolling Bearing Vibrations and Its Application to Failure Identification

**DOI:** 10.3390/s20102964

**Published:** 2020-05-23

**Authors:** Qing Zhang, Tingting Jiang, Joseph D. Yan

**Affiliations:** 1 School of Mechanical Engineering, Xi’an Jiaotong University, Xi’an 710049, China; jtt3118101214@stu.xjtu.edu.cn; 2 Key Laboratory of Education Ministry for Modern Design and Rotor-Bearing System, Xi’an Jiaotong University, Xi’an 710049, China; 3 Department of Electrical Engineering and Electronics, The University of Liverpool, Liverpool L69 3GJ, UK; yaneee@liverpool.ac.uk

**Keywords:** phase synchrony, vibration signal, failure identification, bearing

## Abstract

As the failure-induced component (FIC) in the vibration signals of bearings transmits through housings and shafts, potential phase synchronization is excited among multichannel signals. As phase synchrony analysis (PSA) does not involve the chaotic behavior of signals, it is suitable for characterizing the operating state of bearings considering complicated vibration signals. Therefore, a novel PSA method was developed to identify and track the failure evolution of bearings. First, resonance demodulation and variational mode decomposition (VMD) were combined to extract the mono-component or band-limited FIC from signals. Then, the instantaneous phase of the FIC was analytically solved using Hilbert transformation. The generalized phase difference (GPD) was used to quantify the relationship between FICs extracted from different vibration signals. The entropy of the GPD was regarded as the indicator for quantifying failure evolution. The proposed method was applied to the vibration signals obtained from an accelerated failure experiment and a natural failure experiment. Results showed that phase synchronization in bearing failure evolution was detected and evaluated effectively. Despite the chaotic behavior of the signals, the phase synchronization indicator could identify bearing failure during the initial stage in a robust manner.

## 1. Introduction

Rolling bearings, which support rotating components such as shafts or axles and transfer radial and axial loads in the case of minimized friction, are one of the most widely used functional units in rotating machinery. While a bearing is in operation, vibrations are produced in relatively moving parts including outer races, rolling elements, cages and inner races. The information related to the operating states of parts is contained in the vibration signals and transmits through housings and shafts. In general, acceleration sensors are placed on the bearing housings to collect the vibration signals, and several signal processing methods have been proposed to extract state characteristics from the vibration signals. These state characteristics indicate the occurrence of failure and are used for avoiding breakdown caused by the failure of rolling bearings [1]. However, the complex components of these vibration signals and unstable working conditions of bearings are major problems which negatively influence the accuracy of failure identification, especially in the initial stage of bearing failure.

While a localized defect in a relatively moving part of a rolling bearing is formed and makes contact under load with the surface of another part, a transient impulse is generated in the vibration signal. If a rolling bearing rotates at a constant speed, such impulses will be excited periodically or quasi-periodically [2]. The frequency of these impulses is called the fault characteristic frequency (FCF). As the duration of each impulse is extremely short compared to the interval between impulses, the energy of these impulses is distributed in a very low frequency region over a wide range of frequencies [3]. In this case, characteristics of bearing failure are easily disturbed by vibration components; these are excited by other mechanical elements, and difficult to identify from the spectrum of vibration signals. Fortunately, these impulses may also induce resonance in bearings, adjacent mechanical components, and measuring systems. Amplitude modulation phenomena, caused by carrier waves at the resonant frequency and modulating signals at the FCF, are usually observed in the vibration signals. These resonant signals are concentrated in some relatively narrow frequency regions; they are much higher than the frequency components directly generated by mechanical elements. Therefore, the signal components in resonant frequency regions are regarded as proper processed objects, and demodulation of vibration signals with envelope analysis is performed to extract the characteristics indicating fault impulses. As several resonances may be excited, the major challenge in applying envelope analysis is the selection of the optimal frequency band for demodulation. Fast kurtogram [4] and its improved methods [5,6,7] have been proposed to address this problem. Through demodulation in the well-chosen frequency band, the frequency components at the FCF and their harmonics are clearly identified in the envelope spectrum. Though the defective parts of bearings can be identified through the corresponding FCF, a definite relationship between the envelope spectrum components and failure evolution remains to be established. Moreover, it is disadvantageous for tracking the developing failure during the actual monitoring of bearings.

In practice, many nonlinear factors, such as varying compliance, radial clearance, surface waviness and Hertzian contact forces, commonly influence vibrations produced by defective bearings [8,9]. Therefore, the vibration signals, as the response of nonlinear systems, inevitably exhibit chaotic behaviors. It means that failure characteristics, which are usually the amplitude and phase at the FCF, are sensitive to the working conditions and stochastic at any given moment. Therefore, failure evolution can hardly be estimated by analyzing a certain signal. To overcome this problem, synchrony analysis of vibration signals acquired from different locations or directions has emerged as a method for failure identification. While the impulses excited by a defective bearing transmit through different paths and are collected by sensors, some relative components would appear in multiple vibration signals synchronously or with a fixed delay. Following heightened impulses in the defective contact area, the synchronization of vibration components would become more prominent. Based on this principle, some failure evolution indicators, such as the full spectral features of multivariate empirical mode decomposition (EMD) [10] and vertical–horizontal synchronized root mean square [11], were used to reduce the influence of unstable working conditions and quantify the failure degree. Previous studies focused on the synchrony between the amplitudes of different vibration components owing to its advantages of being intuitive and facilitating easy calculation. However, according to the synchronization theory of nonlinear systems, phase synchrony would occur earlier and more steadily than amplitude synchrony during stimulated oscillations [12,13]. Therefore, phase synchrony analysis is extremely advantageous for bearing failure identification in the initial failure stage and under unstable working conditions.

Phase synchrony analysis (PSA) plays an important role in decoding nonlinear system behavior as well as information transfer mechanisms [14]. It is widely used to discover potential relationships among multivariate signals, especially neurophysiological signals. Several applications, such as functional magnetic resonance imaging analysis [15], new-born electroencephalogram analysis [16], and epilepsy diagnosis [17], have exhibited the capacity of PSA for identifying states considering chaotic signals. However, traditional PSA cannot be directly utilized to process bearing vibration signals. On the one hand, Hilbert transformation is the essential tool to analytically solve signal phases, but it is not suitable for phase calculation of multicomponent or broadband signals [18]. In bearing vibration signals, the failure-induced component (FIC) mixes with background noise and other components; it has two main patterns comprising multiplicative and additive mixtures. Thus, vibration signals become complicated multicomponent signals. On the other hand, the phase is very sensitive to noise. A random fluctuation in signals is likely to cause a jump in phase values. Unfortunately, bearing vibration signals often have a low signal-to-noise ratio. To overcome this problem, a pre-processing stage involving signal decomposition and a robust strategy for synchrony estimation are necessary in the PSA of bearing vibrations.

Therefore, in this study, a PSA method for rolling bearing vibrations was developed. In the pre-processing stage, resonance demodulation and variational mode decomposition (VMD) were combined to separate the FICs from the vibration signals. Then, the instantaneous phase of the FIC was calculated through Hilbert transformation. The generalized difference in instantaneous phases was used to quantify the relationship between FICs extracted from different vibration signals. Finally, the entropy of the generalized phase difference (GPD) was set as the indicator for evaluating phase synchronization and identifying bearing failure. The vibration signals during an accelerated failure evolution and a natural failure evolution were processed using the developed method to verify its capability in identifying bearing failure. The reminder of the paper is organized as follows. Section 2 describes the methodologies and main principle of the PSA method for rolling bearings. Next, case studies demonstrating the applications of this method to determine bearing failure are presented in Section 3. Finally, the conclusions are given in Section 4.

## 2. Method and Principles of PSA

### 2.1. PSA Method for Analyzing Rolling Bearing Vibrations

Phase refers to the position of a periodic signal at a specific moment in the cycle, which is also a measure of the change in the waveform of the signal. It can be described both in the time and frequency domains. If the analyzed signal is non-stationary, like bearing vibrations, the phase is time-varying and termed the instantaneous phase ϕ(t). In this situation, the description of ϕ(t) in the frequency domain is bivariate and difficult to model. Therefore, the phase synchrony in this study was mainly analyzed in time domain.

From the perspective of mathematical simplification, the FIC can be regarded as a mono-component signal, and it mixes with other components having multiplicative and additive forms to constitute the vibration signal. The multiplicative mixture generates the modulations between different signal components. By demodulation, the envelope, which contains the FIC, along with its harmonics and noise, can be separated from the vibration signal. As the amplitude of the FIC is not the subject of concern, sophisticated selection of the demodulation frequency band is no longer required. Theoretically, any resonance frequency band, whose modulating signals contain the FIC, can be used as the demodulation frequency band. For simplicity, a uniform frequency band, located in the resonant frequency region having high energy for most of the analyzed bearing signals, was chosen. After demodulation, the envelope did not change into a mono-component signal; instead, it comprised additive mixtures of the FIC, along with its harmonics and noise. In many experimental or industrial applications, because of the unstable working conditions of bearings, such as uncontrolled fluctuations in shaft speeds, the FIC is not a single-frequency component, but a narrow-band component near the FCF. Therefore, VMD, which decomposes a raw signal into a set of natural oscillatory modes, is applied to self-adaptively and non-recursively separate the FIC from the envelope.

**Figure 1:**
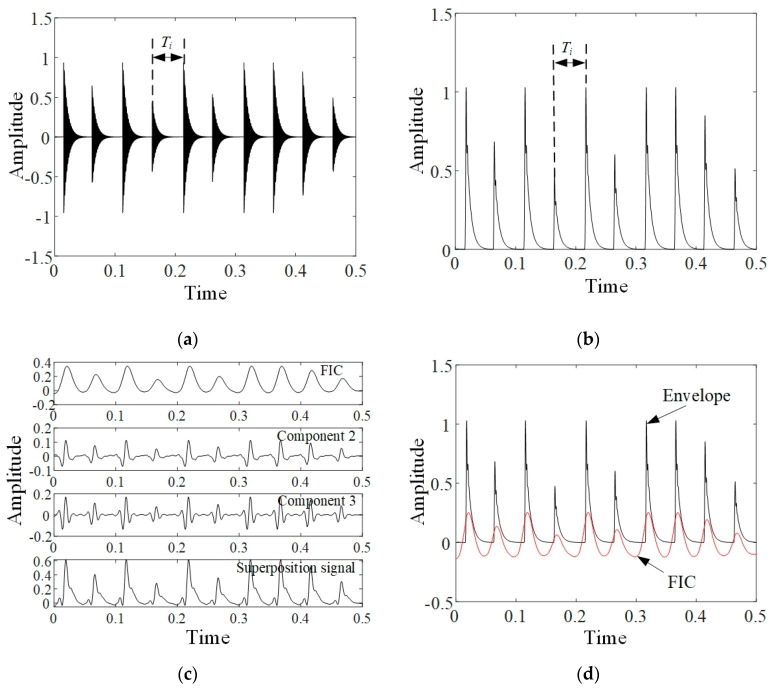
Simulation for pre-processing operations: (**a**) simulated signal; (**b**) envelope of simulated signal; (**c**) signal components decomposed by variational mode decomposition (VMD) and their superposition; (**d**) envelop and failure-induced component (FIC).

The above pre-processing operations can be illustrated with the simulated amplitude modulation signal shown as Figure 1a. To simulate the stochastic fault impulses, the modulating wave of simulated signal was set as multiple transient impulses with unequal amplitudes, attenuation rates and time intervals. As the impulse amplitude is not considered, the fault characteristic is mainly contained in the time intervals between adjacent impulses, which are denoted as $T_{i},\;i=1,\cdots ,n$. Conventionally, the variable $T_{i}$ is represented by the instantaneous frequency f(t) or the instantaneous phase ϕ(t). Therein, the instantaneous frequency f(t) does not need to be explicitly solved due to the correlation with ϕ(t) as follows:

(1) $$f(t)=\frac{1}{2\pi }\frac{d\phi (t)}{dt}$$

The envelope, demodulated from the simulated signal, is given in Figure 1b. Obviously, the spectrum of envelope would cover a wide range of frequencies. By the concurrent decomposition of VMD, FIC, which is a mono-component signal parameterized with ϕ(t), can be separated from the envelope. As shown in Figure 1c, FIC and two other decomposed components can be superposed to approximate the envelope. Under the condition of ignoring envelope amplitude, the instantaneous phase ϕ(t) can be described just by FIC. To comply with the general form of mono-component signal, FIC was centralized by subtracting the mean value. Finally, FIC obtained by pre-processing operations is exhibited in Figure 1d.

**Figure 2:**
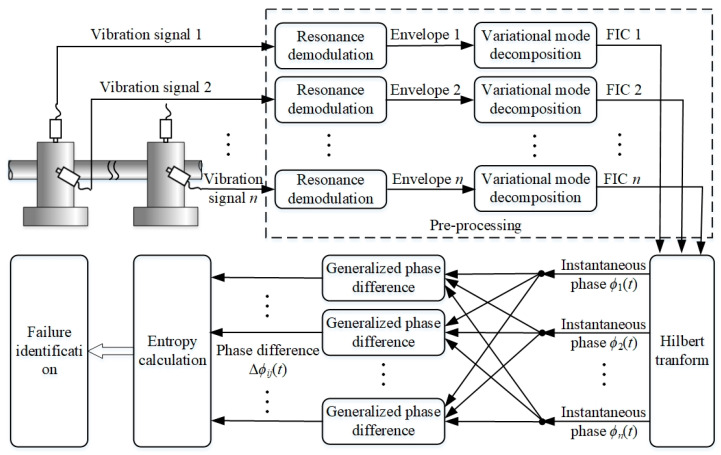
Schematic of phase synchrony analysis (PSA).

Through the pre-processing operations, vibration signals, acquired from different locations or directions, were decomposed to obtain the mono-component FICs. The Hilbert transform was used to solve the instantaneous phases of FICs. Then, the GPDs between each FIC were calculated. When bearings function normally, the FICs should be random and their GPDs follow an approximate uniform distribution between 0 to 2π. Conversely, when failure has occurred and evolved, the instantaneous phases of FICs are locked with each other, and the distribution range of their GPDs is compressed. According to the distribution characteristics, the entropy values of the GPDs are calculated to evaluate phase synchronization and identify failure. The schematic of the proposed method is shown in Figure 2, and detailed descriptions of some operations are provided in the following paragraphs.

### 2.2. Resonance Demodulation

Resonance demodulation is one of the fundamental tools for extracting bearing fault characteristics. It comprises two steps including band-pass filtering and Hilbert transformation. During band-pass filtering, one of the resonance frequency bands is selected in advance. Using an analogue or a digital filter, signal components in this frequency band are retained and other components are removed as much as possible. Then, Hilbert transformation is employed to construct the analytic signals of the components passing through the filter. The modulus of the analytic signal is the envelope, i.e., the modulating signal of the resonance components. Because of the complexity of vibration signals, the envelope still contains the FIC, along with its harmonics and other noise.

The frequency band of the band-pass filter, which is commonly selected by spectral kurtosis or experience, is important for effective noise cancellation. The spectral kurtosis is a fourth-order normalized cumulants that can be used to reveal the amplitude distribution of the impulsive components in the frequency domain. According to the definition of spectral kurtosis [4], the more the impulsive component is concentrated in a certain frequency band, the higher the spectral kurtosis of the filtered signal will be. This means the amplitude of the impulse component will be dominant in the envelope spectrum if the frequency band corresponding to maximization of the spectral kurtosis is selected. Therefore, the optimal frequency band can be determined by searching for the frequency band corresponding to maximization of the spectral kurtosis among all the resonant frequency bands. However, the optimal frequency band is not constant. Instead, it varies with failure evolution owing to the chaotic behavior of the FIC signals. This causes difficulties in continuously and uniformly extracting bearing fault characteristics.

As phase synchrony is more robust than amplitude synchrony, the requirement for selecting the demodulation frequency band is considerably reduced in our method. The central frequency of the demodulation frequency band is selected by experience. According to the existing methods for analyzing bearing vibration signals, a shared high-energy region in spectrums is located, and its center is roughly estimated as the central frequency. Based on an earlier study [19], the bandwidth of the demodulation frequency is selected as the third harmonic of the FCF. This means the FIC and no more than two harmonic components will be included in the demodulated envelope.

### 2.3. Variational Mode Decomposition

VMD, proposed by Dragomiretskiy et al. [20], is an algorithmic method to decompose additive mixture signals into a number of principal modes, which are band-limited intrinsic mode functions. Compared to some recursive decomposition methods, such as empirical mode decomposition and local mean decomposition, VMD effectively reduces mode mixing using a concurrent decomposing process. As it shows excellent performance irrespective of harmonic frequencies and noise, VMD is widely applied to extract fault characteristics of machinery [21,22,23].

The goal of VMD is to decompose a multi-component signal s into *K* band-limited intrinsic modes $\left\{u_{k}\right\}:\left\{u_{1},u_{2},\cdots ,u_{K}\right\}$, whose center frequencies are $\left\{\omega_{k}\right\}:\left\{\omega_{1},\omega_{2},\cdots ,\omega_{K}\right\}$. To achieve the sparsity prior of each mode, a constrained optimization problem is constructed to minimize the sum of the estimated mode bandwidths as follows:

(2) $$\begin{array}{l} \underset{\left\{u_{k},\omega_{k}\right\}}{\min}\left\{\sum_{k}{\left\|\partial_{t}\left[\left(\delta (t)+\frac{j}{\pi t}\right)*u_{k}(t)\right]e^{-j\omega_{k}t}\right\|}_{2}^{2}\right\}\\ s.t.\;\sum_{k}u_{k}=s \end{array}$$

where δ(t) is the Dirac distribution, and ∗ is the convolution operator. To address the minimization problem, a quadratic penalty term and Lagrangian multipliers are introduced to render the problem unconstrained. Then, the augmented Lagrangian is constructed as follows:

(3) $$L(\{u_{k}\},\{\omega_{k}\},\lambda )=\alpha \sum_{k}{\left\|\partial_{t}\left[\left(\delta (t)+\frac{j}{\pi t}\right)*u_{k}(t)\right]e^{-j\omega_{k}t}\right\|}_{2}^{2}+{\left\|s(t)-\sum_{k}u_{k}(t)\right\|}_{2}^{2}+\langle \lambda (t),s(t)-\sum_{k}u_{k}(t)\rangle$$

where α is the penalty coefficient and λ denotes Lagrangian multipliers. The alternation algorithm of multipliers, which constantly updates each intrinsic mode and its center frequency, is used to search the saddle point in Equation (3), which provides the optimal solution to the original problem. The update functions in the frequency domain are given as follows:

(4) $${\hat{u}}_{k}^{n+1}(\omega )=\frac{\hat{s}(\omega )-\sum_{i\neq k}{\hat{u}}_{i}(\omega )+\frac{\hat{\lambda }(\omega )}{2}}{1+2\alpha (\omega -\omega_{k})}$$

(5) $$\omega_{k}^{n+1}(\omega )=\frac{\int_{0}^{\infty }\omega {\left|{\hat{u}}_{k}(\omega )\right|}^{2}d\omega }{\int_{0}^{\infty }{\left|{\hat{u}}_{k}(\omega )\right|}^{2}d\omega }$$

The complete optimization process of VMD can be summarized as follows:

(1) Initialize ${\hat{u}}_{k}^{1}$, ${\hat{\omega }}_{k}^{1}$, ${\hat{\lambda }}^{1}$, and n←0.
(2) Update ${\hat{u}}_{k}^{n+1}$ and $\omega_{k}^{n+1}$ according to Equations (4) and (5).
(3) Perform a dual ascent operation for all ω>0 as
  (6) $${\hat{\lambda }}^{n+1}(\omega )\leftarrow {\hat{\lambda }}^{n}(\omega )+\tau \left(\hat{s}(\omega )-\sum_{k}{\hat{u}}_{k}^{n+1}(\omega )\right)$$
(4) Repeat steps 2 and 3 until the following convergence condition is satisfied.
  (7) $${\sum_{k}\left\|{\hat{u}}_{k}^{n+1}-{\hat{u}}_{k}^{n}\right\|}_{2}^{2}/{\left\|{\hat{u}}_{k}^{n}\right\|}_{2}^{2}<\varepsilon$$

The penalty coefficient α and the number of decomposed modes *K* are the key parameters of VMD, which should be chosen in advance. In VMD, α reflects the compromise between noise suppression and mode duplication. In our method, it was set as 2000 based on experience. Compared to α, *K* has more direct and important effects on the decomposition results. Fortunately, it can be determined using prior information provided in the procedure of resonance demodulation. Because the bandwidth of the demodulation frequency band is approximately triple the FCF, the expected modes, which are decomposed from the envelope by VMD, correspond to the FIC, and the second and third harmonics of the FIC. Therefore, *K* can be set as three or four. If the FIC and its harmonics are dominant in the envelope spectrum, setting *K* as three is appropriate. On the contrary, if the interfering component caused by other noise cannot be ignored in the envelope spectrum, *K* should be set as four.

Among the decomposed modes, a mono-component or band-limited FIC, whose centre frequency is near the FCF, commonly has the highest energy. Based on this property, the FIC can easily be extracted from the decomposition results of VMD.

### 2.4. PSA

After the pre-processing step including resonance demodulation and VMD, the mono-component FICs are extracted from the bearing vibration signals. Suppose $x_{i}(t)$ is the FIC obtained from the *i*-th vibration signal; then, its instantaneous phase $\phi_{i}(t)$ is solved through Hilbert transformation. The Hilbert transform of $x_{i}(t)$ is defined as

(8) $$y_{i}(t)=\frac{1}{\pi }P\int_{-\infty }^{+\infty }\frac{x_{i}(\tau )}{t-\tau }d\tau$$

where P is the Cauchy principal value. Then, the analytic representation of $x_{i}(t)$ is as follows:

(9) $$z_{i}(t)=x_{i}(t)+jy_{i}(t)$$

As $z_{i}(t)$ is a complex signal, its phase, calculated as follows, is defined as the instantaneous phase of $x_{i}(t)$.

(10) $$\phi_{i}(t)=\arctan\left[\frac{y_{i}(t)}{x_{i}(t)}\right]$$

According to the universal concept of phase synchronization, the phenomenon of phase locking is observed when different signal components are induced by the same stimulation. The locking condition is given as follows:

(11) $$\left|\phi {\left(t\right)}_{i,j}\right|<const,\;where\;\phi {\left(t\right)}_{i,j}=n\phi_{i}(t)-m\phi_{j}(t)$$

where n and m are integers indicating the possible locking pattern. Based on the locking condition, the GPD between two instantaneous phases is defined as follows:

(12) $$\Delta \phi {\left(t\right)}_{ij}=\left|n\phi_{i}(t)-m\phi_{j}(t)\right|$$

For any pair of analyzed vibration signals, FICs excited by the same failure have a similar frequency distribution. Therefore, only the 1:1 locking condition, i.e., the case where n=m=1, is considered. As a result, GPD is rewritten as

(13) $$\Delta \phi {\left(t\right)}_{ij}=\left|\phi_{i}(t)-\phi_{j}(t)\right|\mathrm{mod}\;2\pi$$

where mod 2π is used to address noise-induced phase jumps.

To characterize the strength of phase synchronization, the deviation of the actual distribution of the GPD from the uniform distribution is quantified. The distribution range of the GPD is divided into *N* bins. The probability of $\Delta \phi {\left(t\right)}_{ij}$ being in the *k*-th bin is denoted as $p_{k}$. Then, the Shannon entropy of the GPD distribution is defined as follows:

(14) $$S_{ij}=\sum_{k=1}^{N}p_{k}\ln p_{k}$$

An indicator of phase synchronization is constructed according the normalized entropy as follows:

(15) $$\rho_{ij}=\frac{S_{\max}-S_{ij}}{S_{\max}}$$

where $S_{\max}=\ln N$ is the maximum entropy, which is obtained in the case of uniform distribution. Normalized in this manner, $0\leq \rho_{ij}\leq 1$, where $\rho_{ij}=0$ corresponds to a uniform distribution and $\rho_{ij}=1$ corresponds to a Dirac distribution. This also means that, if $\rho_{ij}=0$, there is no phase synchronization between $x_{i}(t)$ and $x_{j}(t)$ at all. On the contrary, if $\rho_{ij}=1$, $x_{i}(t)$ and $x_{j}(t)$ have the perfect relationship with phase synchronization. The actual value of $\rho_{ij}$ is in the range of 0–1 and is capable of quantifying the phase synchrony.

Using the proposed method, bearing vibration signals acquired from different locations or directions are pre-processed to extract mono-component FICs. Then, the indicator $\rho_{ij}$ is constructed to quantitatively evaluate the phase synchronization between each pair of FICs and used to identify the bearing failure.

## 3. Application Case Studies

To demonstrate the ability of our method to evaluate phase synchronization and identify failure, this section presents two application cases involving rolling bearing failure. Both the cases have complete life cycles starting from normal functioning to degradation, involving two failure modes: accelerated failure and natural failure.

### 3.1. Application to Accelerated Bearing Failure

**Figure 3:**
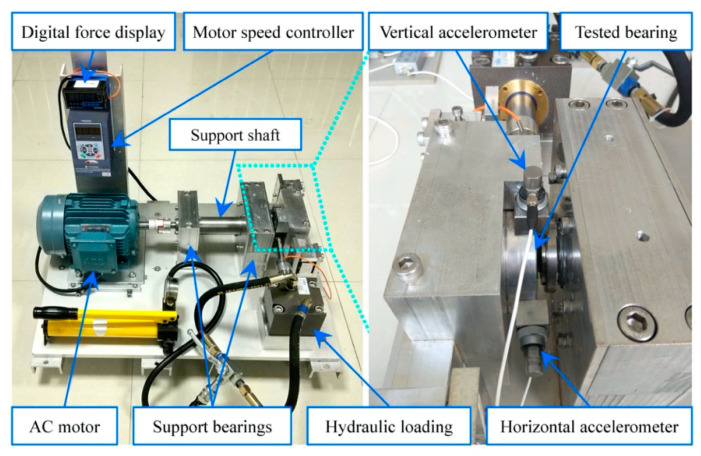
Layout of the test rig used for accelerated failure.

The accelerated failure data were acquired from the test rig shown in Figure 3 and they are part of the XJTU-SY bearing datasets [24]. The LDK UER204 type rolling bearing was tested, and two PCB 352C33 accelerometers were placed on the bearing housing in horizontal and vertical directions. The radial force was provided by a hydraulic loading system to generate the condition for accelerated failure. Vibration signals were recorded at a sampling frequency of 25.6 kHz and collected with an interval of 1 min.

The dataset marked Bearing 1_3 was selected as the analysis object. It comprised 158 groups of data, including horizontal and vertical vibration signals. Corresponding to this dataset, the tested bearing was operated under a radial force of 12 kN and rotating speed of 2100 rpm. After running for 2 h and 38 min, the bearing broke down with the failure of the outer race. In the dataset, signals within 0.5 s were truncated as the analyzed sample. Because the FCF of the outer race was 107.91 Hz, the frequency band between 800 and 1100 Hz was selected by experience for resonance demodulation. In VMD, the number of decomposed modes *K* was set as four. The band-limited intrinsic mode, which has the maximum root mean square (RMS) among the decomposed modes of VMD, was regarded as the FIC. Then, the instantaneous phase of the FIC was calculated, and phase synchrony was quantitatively evaluated.

**Figure 4:**
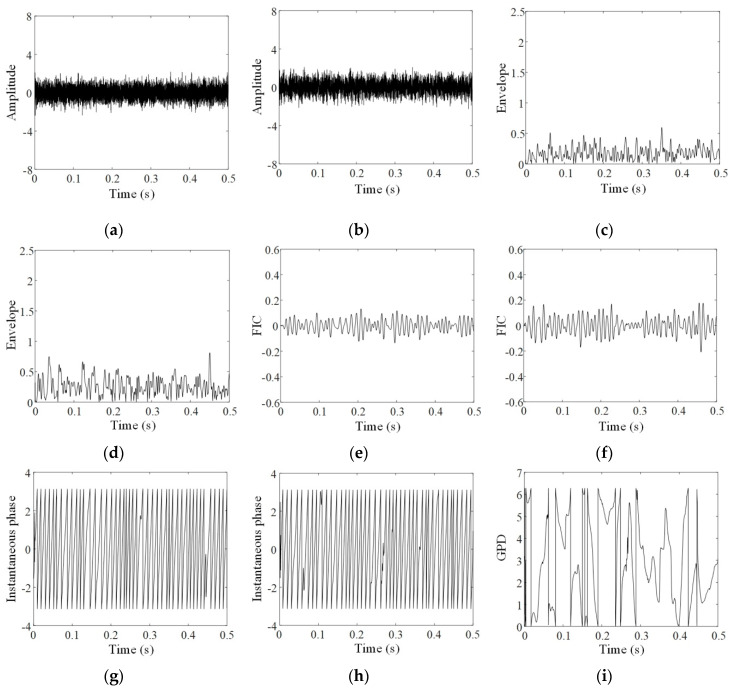
PSA of the 60th minute vibration signals: (**a**) horizontal signal; (**b**) vertical signal; (**c**) envelope of horizontal signal; (**d**) envelope of vertical signal; (**e**) FIC of horizontal signal; (**f**) FIC of vertical signal; (**g**) instantaneous phase of horizontal FIC; (**h**) instantaneous phase of vertical FIC; (**i**) generalized phase difference (GPD) of vibration signals.

**Figure 5:**
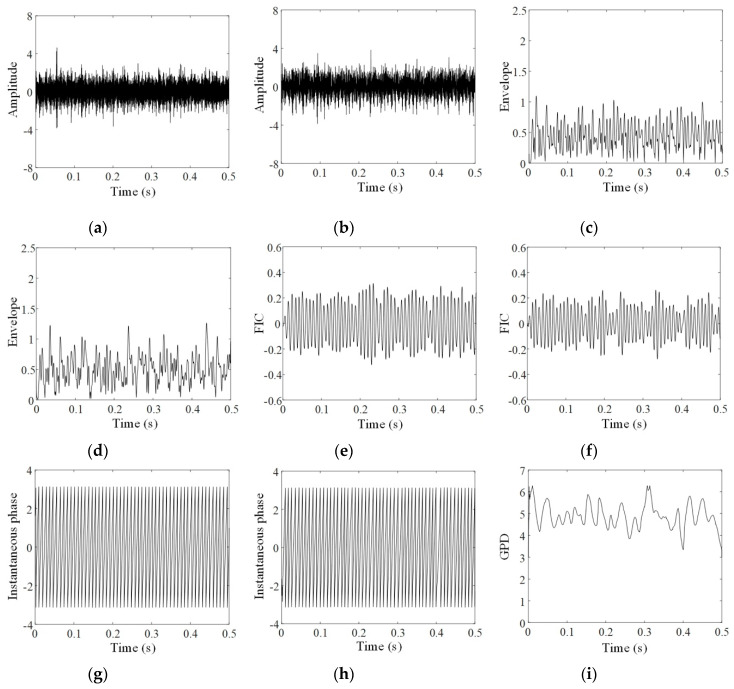
PSA of the 90th minute vibration signals: (**a**) horizontal signal; (**b**) vertical signal; (**c**) envelope of horizontal signal; (**d**) envelope of vertical signal; (**e**) FIC of horizontal signal; (**f**) FIC of vertical signal; (**g**) instantaneous phase of horizontal FIC; (**h**) instantaneous phase of vertical FIC; (**i**) GPD of vibration signals.

**Figure 6:**
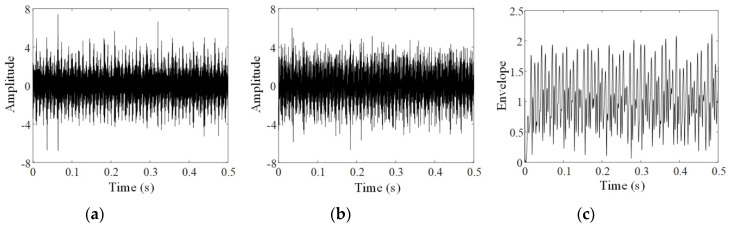

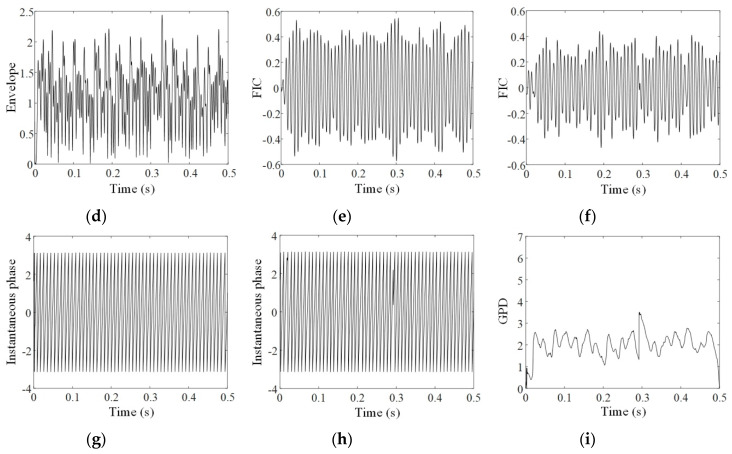
PSA of the 120th minute vibration signals: (**a**) horizontal signal; (**b**) vertical signal; (**c**) envelope of horizontal signal; (**d**) envelope of vertical signal; (**e**) FIC of horizontal signal; (**f**) FIC of vertical signal; (**g**) instantaneous phase of horizontal FIC; (**h**) instantaneous phase of vertical FIC; (**i**) GPD of vibration signals.

To present the intermediate results of each step in the proposed method, the analyses of the three groups of data, respectively acquired at the 60th, 90th, and 120th minutes, are given in Figure 4, Figure 5 and Figure 6. In these figures, a and b respectively show original signals in horizontal and vertical directions; c and d display the envelopes of original signals obtained by resonance demodulation, while e and f exhibit the FICs separated from the envelope, g and h provide the instantaneous phases of the FICs, and i gives the GPD between two instantaneous phases of horizontal and vertical signals.

Comparing Figure 4, Figure 5 and Figure 6 shows that FICs continuously increase and their instantaneous phases become regular with the increasing experimental period. As shown in Figure 4g,h, the instantaneous phases at the 60th minute show several aberrations with uneven spacing. As a result, the GPD shown in Figure 4i is randomly distributed between 0 and 2π. On the contrary, the instantaneous phases at the 120th minute, illustrated in Figure 6g,h, are approximately regular triangle waves. As shown in Figure 6i, most of the GPDs between these two instantaneous phases are limited in the range of [0.5π,0.8π]. The phenomenon of phase locking is clearly observed.

**Figure 7:**
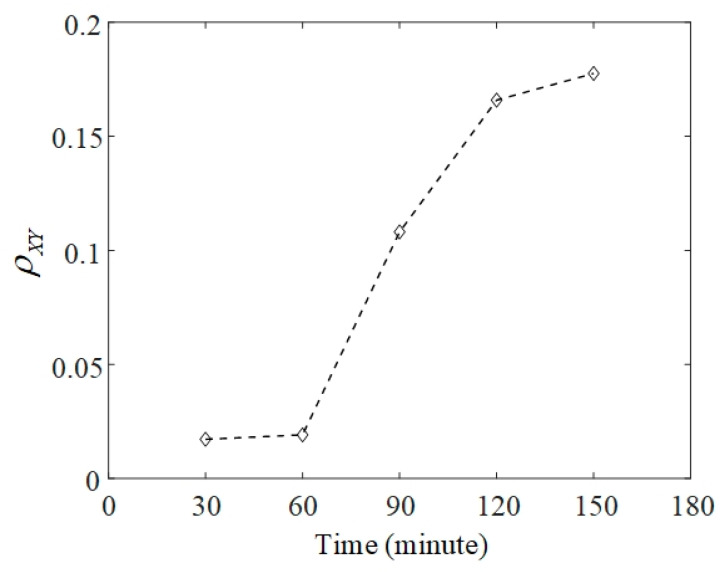
Phase synchronization indicator for accelerated failure.

To track the failure evolution, vibration signals were selected from the Bearing 1_3 dataset every 30 min to calculate the phase synchronization indicator between horizontal and vertical signals. This indicator is denoted as $\rho_{XY}$, and the curve of $\rho_{XY}$ is portrayed in Figure 7. At the 30th and 60th minutes, $\rho_{XY}$ values are 0.017 and 0.019, respectively. It means that there is almost no synchronization phenomenon at the early stage of the experiment. Corresponding to the signals at the 90th minute, $\rho_{XY}$ quickly increases to 0.108. A stable phase-locking relationship was established between horizontal and vertical signals at this moment. This can be observed in Figure 5i. As the experiment time increased, $\rho_{XY}$ reached 0.166 and 0.178, respectively, at the 120th and 150th minutes. Finally, failure at the outer race was confirmed at the 158th minute. This result verified that the indicator of phase synchronization could satisfy the demand for quantitative assessment of failure evolution in bearings.

### 3.2. Application to Natural Failure of Bearing

**Figure 8:**
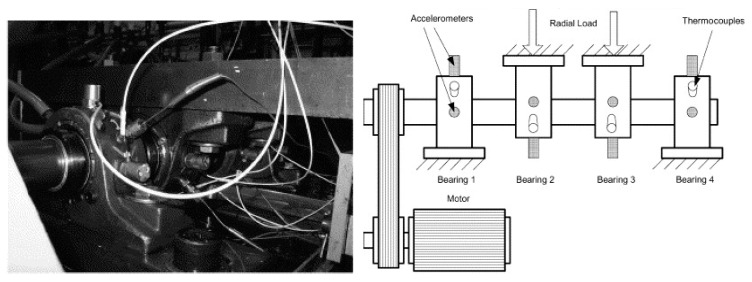
Bearing test rig for analyzing natural bearing failure.

The natural failure data of bearings was provided by the Intelligent System Maintenance Center of the University of Cincinnati [25]. A test rig, shown as Figure 8, was used to perform bearing run-to-failure tests under normal running conditions, corresponding to a radial load of 26.67 kN and rotating speed at 2000 rpm. A magnetic plug was installed in the oil pipe to collect debris from the oil as evidence of bearing failure. Vibration signals were acquired at a sampling frequency of 20 kHz and the data length was 20,480 during each sampling.

**Figure 9:**
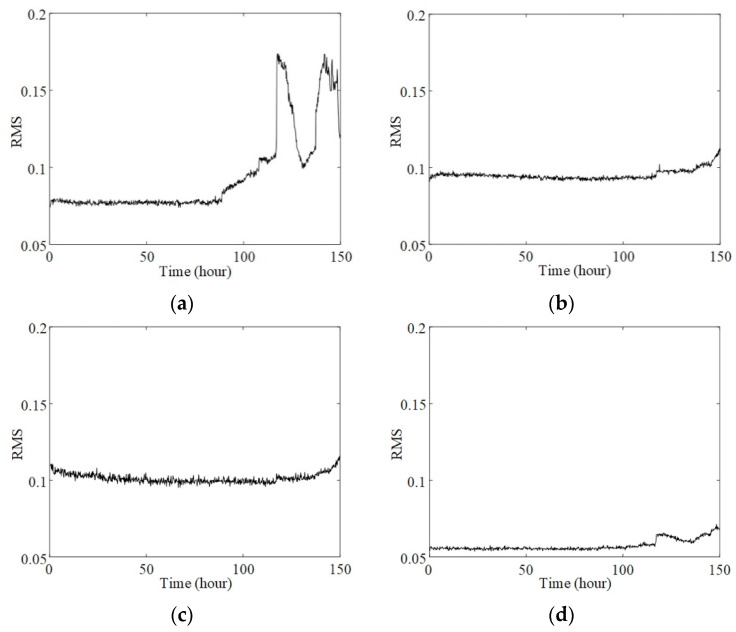
Root mean square (RMS) of vibration signals: (**a**) bearing 1; (**b**) bearing 2; (**c**) bearing 3; (**d**) bearing 4.

The dataset 2#, comprising four channels of vibration signals strictly collected every 10 min, was used to verify the proposed method. Corresponding to the dataset, the tested bearing ran for a total of 164 h before an outer race failure on bearing 1 was confirmed. Considering the significance of initial failure identification and serious uncertainty of failure data near the end of the experiment, the vibration signals in the first 150 h were selected to analyze the behavior of phase synchrony. The RMS indexes of the selected signals, which reflect the average power of the signal amplitudes, are given presented in Figure 9. As the failure occurred on bearing 1, the RMS of bearing 1, given in Figure 9a, dramatically increased after the 115th hour. However, the RMS decreased after approximately 6 h. Such variations cause difficulties in failure identification. As for the vibration signals of other acquisition channels, the RMS indexes just exhibited some weak increments during the experimental process. Therefore, according to Figure 9, synchronization of the RMS cannot be observed.

In reference [11], a synchronized root mean square (SRMS) index was proposed to quantify the synchronization between bearing vibration signals acquired from different directions. Suppose $s_{i}$ and $s_{j}$ are signals of length $N_{s}$, the SRMS between them was defined as

(16) $$SRMS_{i,j}=\sqrt{\frac{\sum_{k=1}^{N_{s}}{\left[s_{i}(k)-{\bar{s}}_{i}\right]}^{2}}{\sum_{k=1}^{N_{s}}{\left[s_{j}(k)-{\bar{s}}_{j}\right]}^{2}}}$$

**Figure 10:**
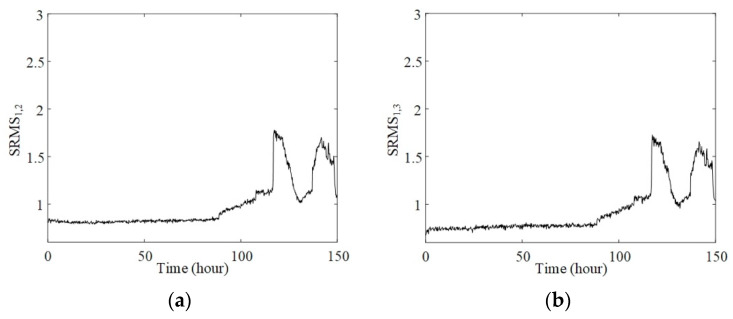

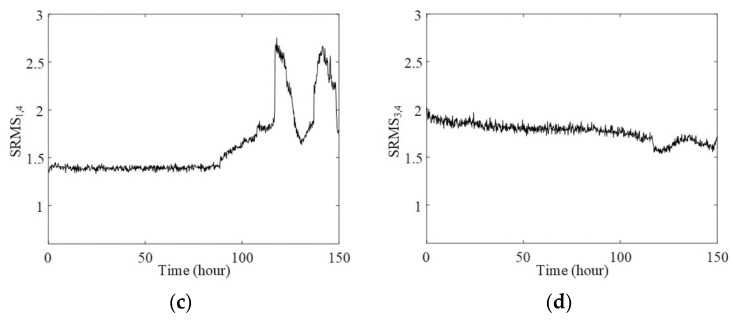
Synchronized root mean square index between (**a**) bearings 1 and 2; (**b**) bearings 1 and 3; (**c**) bearings 1 and 4; (**d**) bearings 3 and 4.

For comparing with the proposed method, the SRMSs between signals from different bearings were calculated and portrayed in Figure 10. Since the RMSs of bearings 2, 3, and 4 did not change significantly during the experimental process, ${\mathrm{SRMS}}_{1,2}$, ${\mathrm{SRMS}}_{1,3}$, and ${\mathrm{SRMS}}_{1,4}$ had similar shapes with the RMS curve of bearing 1. The irregular fluctuations of these SRMS curves, showed as Figure 10a through 10c, make it difficult to track failure evolution. In addition, the ${\mathrm{SRMS}}_{3,4}$ shown in Figure 10d also fails to provide clear information of failure that occurred on bearing 1.

**Figure 11:**
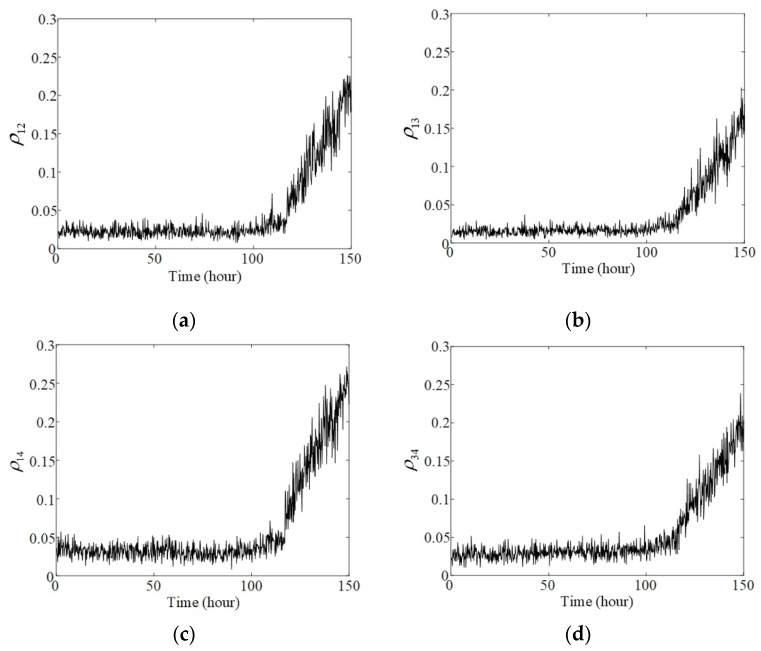
Phase synchronization indicator between (**a**) bearings 1 and 2; (**b**) bearings 1 and 3; (**c**) bearings 1 and 4; (**d**) bearings 3 and 4.

The proposed method was used to analyze the dataset. According to the spectral distribution and FCF of the outer race, the demodulation frequency band was set between 730 and 1500 Hz. The number of decomposed modes of VMD was set as three. The FIC was separated from each original signal, and four groups of phase synchronization indicators between signals from different channels at the same collecting moment, denoted as $\rho_{12}$, $\rho_{13}$, $\rho_{14}$, and $\rho_{34}$, were calculated. The results are shown in Figure 11.

All the phase synchronization indicators show stable increasing trends after the 115th hour. Although bearings 2, 3, and 4 did not fail, the vibration signals acquired from their housings still maintained phase synchronization with the signal of bearing 1. Moreover, signals acquired from bearings 3 and 4, situated away from the fault bearing, also exhibited similar phase synchrony, as shown in Figure 11d. These results prove that the FIC can transmit through housings and shafts and induce phase synchronization along the transmission path. The phase synchronization indicators obtained using the proposed method are capable of detecting and evaluating the potential synchronization. This method provides a new approach for identifying and tracking bearing failure by fusing multichannel vibration signals.

## 4. Discussion and Conclusions

Owing to the chaotic behaviors of bearing vibration signals, it is difficult to identify bearing failure by analyzing a certain signal of a single channel. Thus, a novel method based on the phase synchrony between multiple signals sensed from the vibration transmission path is proposed to track failure evolution. The proposed method has two significant features as follows:

(1) A mono-component FIC is extracted from vibration signals through a pre-processing step including resonance demodulation and VMD. The interference components mixed in multiplicative and additive forms are removed to obtain an optimal band-limited FIC, whose instantaneous phase can be solved using Hilbert transformation.
(2) The indicator, which is the entropy of the GPD between FICs, is constructed to quantitatively evaluate phase synchronization of vibration signals. Despite the chaotic behavior of the signals, the phase synchronization indicator could identify bearing failure during the initial stage in a robust manner.

This method was successfully applied for analyzing two bearing datasets, which represent the bearing’s life cycle experiments of the bearing with accelerated and natural failure. The results showed that bearing failure induced phase synchronization in vibration signals and the proposed method could effectively evaluate phase synchronization to track failure evolution. 

Extracting the mono-component or band-limited FIC is a crucial step of PSA. Although the selection requirements for the parameters used in resonance demodulation and VMD are significantly reduced, there are still several challenges and practical problems requiring further investigation. Firstly, in different failure stages, variable parameters may be more suitable for accurate decomposition of the FIC than the constant parameters. Secondly, endpoint effects of VMD cause some uncontrolled errors in the GPD. Thirdly, the effects of sampling frequency and data length have not been considered. We intend to focus on these aspects in our future studies.
